# Hyper-Stain Inspector: A Framework for Robust Registration and Localised Co-Expression Analysis of Multiple Whole-Slide Images of Serial Histology Sections

**DOI:** 10.1038/s41598-017-05511-w

**Published:** 2017-07-17

**Authors:** Nicholas Trahearn, David Epstein, Ian Cree, David Snead, Nasir Rajpoot

**Affiliations:** 1 Department of Computer Science, University of Warwick, Coventry, United Kingdom; 2 Mathematics Institute, University of Warwick, Coventry, United Kingdom; 3 International Agency for Research on Cancer, World Health Organization, Lyon, France; 4 Department of Pathology, University Hospitals Coventry and Warwickshire, Coventry, United Kingdom

## Abstract

In this paper, we present a fast method for registration of multiple large, digitised whole-slide images (WSIs) of serial histology sections. Through cross-slide WSI registration, it becomes possible to select and analyse a common visual field across images of several serial section stained with different protein markers. It is, therefore, a critical first step for any downstream co-localised cross-slide analysis. The proposed registration method uses a two-stage approach, first estimating a fast initial alignment using the tissue sections’ external boundaries, followed by an efficient refinement process guided by key biological structures within the visual field. We show that this method is able to produce a high quality alignment in a variety of circumstances, and demonstrate that the refinement is able to quantitatively improve registration quality. In addition, we provide a case study that demonstrates how the proposed method for cross-slide WSI registration could be used as part of a specific co-expression analysis framework.

## Introduction

Cross-slide analysis is key for many tasks in the field of pathology. Frequently, such analysis is performed on serial or transverse sections taken consecutively from a tissue block. Each tissue section is stained to highlight particular features of interest in the tissue. For cross-slide analysis, immunohistochemical (IHC) stains are of particular interest, as they indicate the presence and expression of a chosen protein. Expression profiles of the various different markers can usually be compared directly, as each section is only a microscopic distance (typically 3–5 microns) from the neighbouring sections. Thus larger anatomical structures are likely to be present across many sections. Analysis of the expression profiles of multiple markers in a common region of interest, such as a tumour region, can potentially reveal important information about the tumour’s molecular composition. Visual analysis is subjective and prone to human error, and in a number of studies these have been shown to result in significant inter- and intra-observer variability^1–3^. This observation demonstrates the need for high-quality automated analysis.

A major challenge for automated multi-slide analysis is that sectioning the sample destroys the 3-dimensional structure, and thus the continuity of the tissue in the z-axis. This means that any co-localised cross-slide analysis is impossible without first re-aligning, or registering, the images. Cross-slide analysis is important as different sections may be stained with different markers, and thus highlight different items of interest. By registering the sections, we can see which markers are co-expressed in the same part of the tissue, which could provide important additional information to the pathologist. Currently the registration and subsequent analysis of slides from serial sections is performed manually by the pathologist, which can be slow, inaccurate, and is difficult to maintain with large image stacks. Therefore, in order to proceed any further with automated analysis of image data from serial sections it is important first to establish a way of automatically registering the whole slide images. Registration must be possible even when there are significant differences between the pair of sections, for the problem of serial section registration this condition is key because it is likely that some of the tissue structures will not persist across multiple sections and thus cannot be used for alignment, this is the case in part due to the section thickness being of a similar order to the depth of the structures themselves. The registration method must also complete in a reasonable amount time, such that it would not interfere with the flow of routine practice.

Following on from registration, any number of cross-slide analysis tasks can be performed. One instance is the automatic scoring of immunohistochemical (IHC) stained breast cancer slides, where we attempt to assess the quantity of the IHC marker’s reaction in a particular region of the tissue. Immunohistochemically stained slides are normally scored manually by the pathologist in order to determine the types of treatment that the patient is likely to respond positively to, allowing for a more tailored treatment program. Each marker has its own unique expression pattern, which may depend on factors such as which organelle in the cell it is likely to be expressed in (e.g. nucleus, cytoplasm, or membrane) and the variation of reactivity. Thus a variety of scoring systems have been established for different IHC markers. One such scoring method is the Allred scoring system^4^ for Estrogren Receptor (ER) and Progresterone Receptor (PR), which specifies numerical values for the intensity and the proportion of tumour nuclei stained with the ER or PR marker. With accurate registration and reliable estimation of stain expression, key features of a set of markers can be assessed automatically, and thus the manual scoring system can be emulated with an automated system.

**Figure 1:**
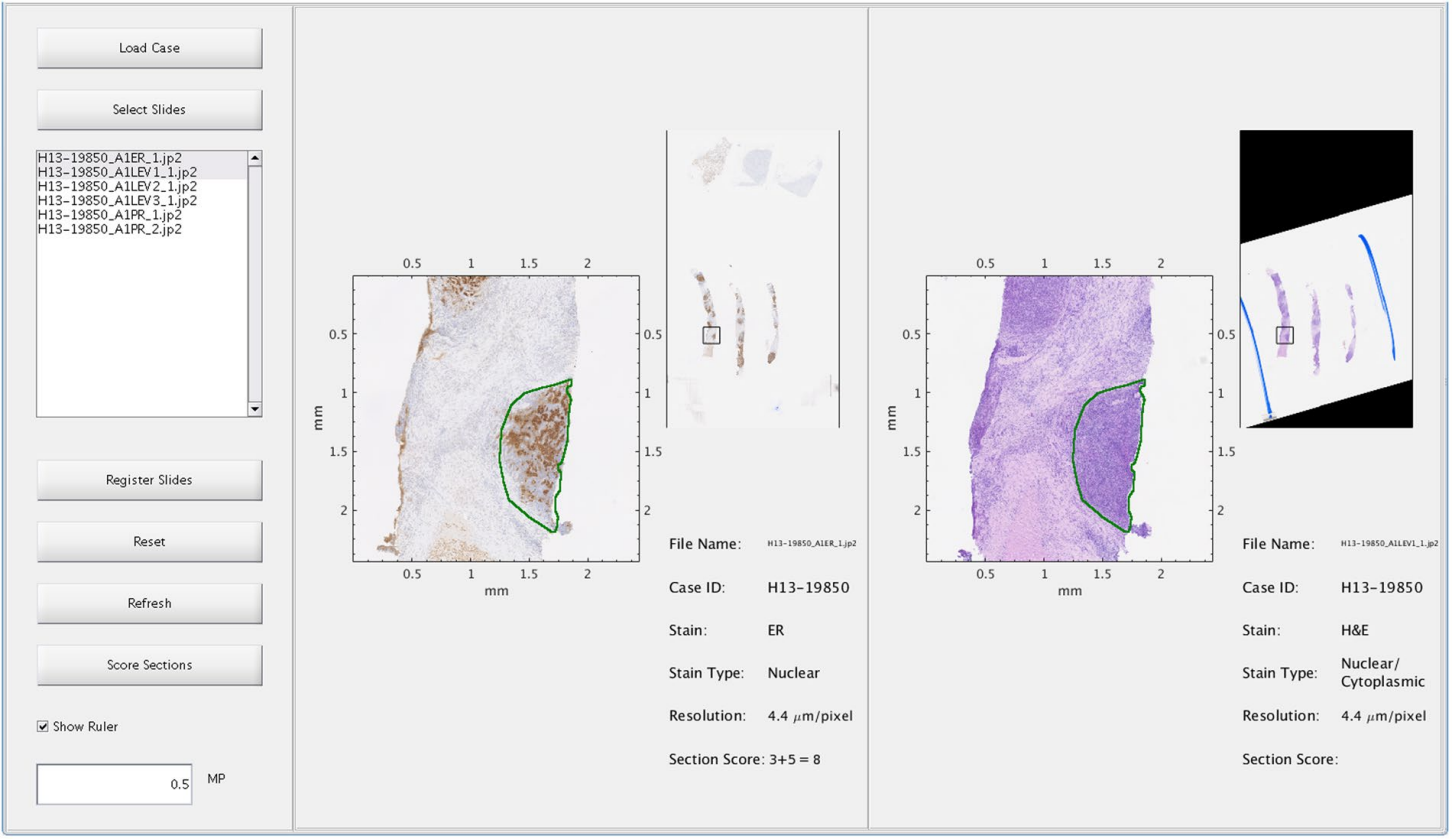
A screenshot of the Hyper-Stain Inspector software. The left panel contains the controls for various functions of the system, such as registration and IHC scoring. This panel also has a list box containing all of the whole slide images within the case, which the user can select or deselect to respectively load or unload the WSI into the system for viewing. Similarly to a slide viewer, loaded slides can be directly navigated to select a visual field for closer inspection. In addition, there are buttons which allow automated algorithms, such as registration and scoring, to be applied. The central and right panels show WSIs that have been loaded for viewing. Each panel contains a close-up viewing area (left), a view of the whole slide (top-right), and any auxiliary information about the slide (bottom-right). The slides shown have been registered and scored, the score is shown in the bottom-right, labelled as “Section score”, in addition, the area that was selected for scoring is highlighted on each image by a green line.

We present a novel framework for robust registration of serial sections stained with different markers, enabling many forms of cross-slide analysis such as localised co-expression tasks. The complete framework has been implemented as a software package, known as the Hyper-Stain Inspector, which is capable of analysis on up to 16 whole slide serial section images at one time. Figure 1 shows an example of registration and ER scoring within the Hyper Stain Inspector environment. Using this software, we demonstrate how the proposed framework may be applied to a specific cross-slide analysis task by providing experimental results of both the registration algorithm and its application to ER/PR scoring. Our findings show that, with the aid of registration, we can achieve simultaneous automated scoring of IHC markers with close agreement to that of a pathologist. We will also detail the individual components of the serial section registration algorithm; specifically an approximate alignment on the external boundaries of the tissue sections and a refinement stage using the sections’ internal tissue structures.

## Literature Review

Most automated registration methods typically fall into one of the following two categories: feature-based methods and intensity-based methods^5^. Intensity-based methods use the pixel intensities of the images directly, typically by finding the transformation that aligns two images, such that their pixel intensities optimise a chosen similarity measure. Feature-based methods instead align the images based upon features that are first extracted from each image, a matching process is applied to find pairs of corresponding features from the two images. These feature pairs are then used to generate the desired registration transformation parameters.

For intensity-based registration, much attention has been given to the use of mutual information (MI)^6–8^, which has also been applied in the context of digital pathology images^9, 10^. MI-based approaches work by finding a transformation that maximises the mutual information between the two aligned images. The assumption is that, when two images are aligned, there will be a clear relationship between their pixel intensities, and thus the mutual information between the two images will be at its maximum value. The strength with this particular similarity measure is that the intensities need only be correlated, not necessarily similar, which allows for the comparison of images of different modalities, such as those with different stain colours. However, each IHC marker highlights the presence of a different antigen, and thus the staining pattern is likely to be different for each marker. This means that there may not be a clear relationship between the pixel intensities of the two IHC WSIs: such a relationship is necessary for accurate MI-based registration. This is a limitation that is likely to exist for all methods that operate on intensity alone. For this reason we have chosen to pursue feature-based approaches in our work.

Point-set registration methods can be categorised within feature registration, where alignment is based on the coordinate locations of the detected features. Iterative Closest Point (ICP)^11^ was an early solution to the point-set registration problem. Each point from the source point set is assigned to its closest point in the target set, one can then generate the transformation that best aligns the assigned point pairs and apply it to the source set. This process is repeated until convergence is achieved. ICP algorithms are prone to stalling in sub-optimal local minima, and thus may not produce a good registration if the initial registration is poor. In addition, convergence is not guaranteed in many cases^12^. Another approach to point-set based registration is Coherent Point Drift^13^, which represents the target point set as a Gaussian Mixture Model (GMM) and attempts to find the deformation that best maps the source point set to the GMM. The optimal transformation is found by Expectation Maximisation optimisation. Methods for different transformation models, such as rigid, affine, and non-linear transformations are specified. This method may still fail to produce an optimal alignment if it reaches an undesirable local minima.

The problem of local minima can be mitigated by selecting an appropriate initial transformation, however this cannot be known ahead of time and thus still needs to be computed. A common solution^14–16^ is to first apply a fast registration algorithm, which produces an approximate alignment that can then be improved upon. One such area is edge-based registration. Edge-based registration methods typically extract the external boundary of a pair of objects, usually modelling them as a set of points on a polygon, and then attempt to register the objects on the basis of just these boundaries. An object’s boundary is a much less data intensive representation of the object when compared with the original image, and thus edge-based registration can often be considerably faster than methods that work on a pixel level. Hierarchical Chamfer Matching^17^ is an example of an edge-based registration method, that attempts to register a pair of boundaries by minimising the chamfer distance between them. A multi-resolution approach is applied to the matching, first aligning at a low resolution and making subsequent refinements on higher resolution representations of the boundaries. The algorithm requires one boundary to be transformed into a distance image, which for higher resolutions is a very costly operation, making it unsuitable for fast registration.

Curvature is also of interest for edge-based alignment, in part because it is a feature that is invariant to rotation and translation. It is also robust to small non-linear distortions, such as those that may occur to serial tissue sections during the processes of slide preparation. Trahearn *et al*.^18^ presented a fast registration method for whole-slide images of serial sections using the Curvature Scale Space representation of a tissue section’s boundary. The CSS representation^19, 20^ is a scale and rotation invariant shape descriptor that represents a curve by tracking extreme points of curvature along the boundary at differing resolutions.

Mueller *et al*.^21^ presented a whole slide registration system, which aligns neighbouring sections using a two-stage spline-based alignment method. The best non-rigid alignment was determined using elastix^22^, an existing open-source registration software. An analysis of the registration accuracy and processing time was provided for a variety of different conditions, such as different image resolutions and rigid vs. non-rigid registration. Magee *et al*.^23^ presented a whole slide registration system for multiple stains, focused on the problem of 3D reconstruction. In such a scenario, the goal is to build a three dimensional volume from two dimensional sections. In this approach the image is categorised into different tissue classes. The resultant probability maps are then aligned using the standard approach of mutual information alignment.

One of the earliest works on automatic IHC quantification was ImmunoRatio by Tuominen *et al*.^24^. The main contribution of the work is a web based system for the analysis of breast cancer images, focusing on slides stained with ER, PR, and Ki67. While the system does not produce the scores themselves, it does provide the pathologist with a number of pieces of key information estimated from the image, such as the percentage of the nuclei stained by IHC. The work shows a clear positive relationship between the statistics calculated with the software and those produced manually, providing strong indication of the potential of automated IHC analysis. ImmunoRatio does not distinguish between the IHC staining of tumour nuclei and non-tumour nuclei however. This limits the power of any analysis as it is often only the staining of the tumour nuclei that is clinically significant. In addition, this work was only focused on single slide analysis. Khan *et al*.^25^ presented an automated method of estimating the ER/PR score of slides, using the H-score scoring system. The paper follows a similar framework to ImmunoRatio, extracting the IHC and Haematoxylin stain intensity from the image and performing analysis upon them. In addition the paper describes a binning process for categorising the stains into four intensity groupings, as is estimated by pathologists when determining the H-score. However the exact value ranges of the bins are not given. There is again no distinction between tumour and non-tumour tissue in this work, which is likely to affect the accuracy of the scores it produces. These methods highlight the need for good tumour segmentation within any scoring framework, as the results that are obtained without taking this into account may be misleading and result in an incorrect treatment being given to the patient. IHC Profiler, by Varghese *et al*.^26^, is an ImageJ plugin, intended to perform automated quantification and analysis on IHC images. The method calculates an H-Score for the region of interest, which it achieves by dividing the image into four groups of IHC stain intensity: negative, low positive, positive, and high positive. The boundaries between stain groups are determined empirically. The binning approaches adopted by this method and Khan’s method are an attempt to emulate the manual scoring protocol that has been adopted by pathologists for routine practice, and is therefore a practical design choice for an automated version. Much like the previously discussed methods, the H-Score is calculated on the whole of the digitally separated DAB channel, rather than just the tumour nuclei, which could give misleading results in situations with stained non-tumour nuclei or residual IHC staining across the tissue.

## Results

### Registration

We have evaluated the quality of the registration across a set of 10 cases, each containing several serial sections to be registered. For a given case, each slide is registered to its neighbour using the proposed framework. Performing this consecutively and combining transformations allows for a case-wide registration. To quantitatively assess the quality of the alignment we calculate the chamfer distance between adjacent sections following alignment, as follows,

1 $$D({B}_{ref},{B}_{reg})=\sum _{s\in {B}_{ref}}\mathop{{\rm{\min }}}\limits_{t\in {B}_{reg}}(\Vert s-t\Vert )$$

where *B*_*ref*_ and *B*_*reg*_ represent the boundaries of the reference and registered tissue section, respectively, each an ordered set of points that define its boundary.

For this experiment we have selected 10 cases. 7 of these cases, labelled as ERPR in Table 1, are breast cases each containing 5 slides. The remaining 3 cases, labelled as LYM in Table 2, are lymph node cases each containing 10 slides. Each slide is scanned at a resolution of 0.275 *μm*/pixel, equivalent to a 40× optical zoom.

**Table 1:** A comparison of the registration of breast cases, each containing a set of 5 slides, before and after refinement.

Case	Average Chamfer Distance	
	Approximate Registration	Approximate & Refinement Registration
ERPR1	333.763	**199**.**997**
ERPR2	315.168	**286.776**
ERPR3	354.245	**213**.**911**
ERPR4	367.631	**206**.**940**
ERPR5	329.960	**191**.**236**
ERPR6	511.227	**278**.**304**
ERPR7	**285**.**998**	299.612
*Average*	*356*.*856*	***239***.***539***

Registration quality is measured as the average Chamfer distance between the external boundaries of registered tissue sections. Slides were of the following markers: ER, PR, HER2, H&E, and Negative Control.

**Table 2:** A comparison of the registration of 10 slide lymph node cases before and after refinement.

Case	Average Chamfer Distance	
	Approximate Registration	Approximate & Refinement Registration
LYM1	347.04	**247**.**851**
LYM2	311.320	**271**.**328**
LYM3	365.789	**268**.**177**
*Average*	*341*.*383*	***262***.***452***

Registration quality is measured as the average Chamfer distance between the external boundaries of registered tissue sections. Slides were of the following markers: BCL2, BCL6, CAM5.2, CD3, CD5, CD10, CD20, Ki67, H&E, and Negative Control.

**Figure 2:**
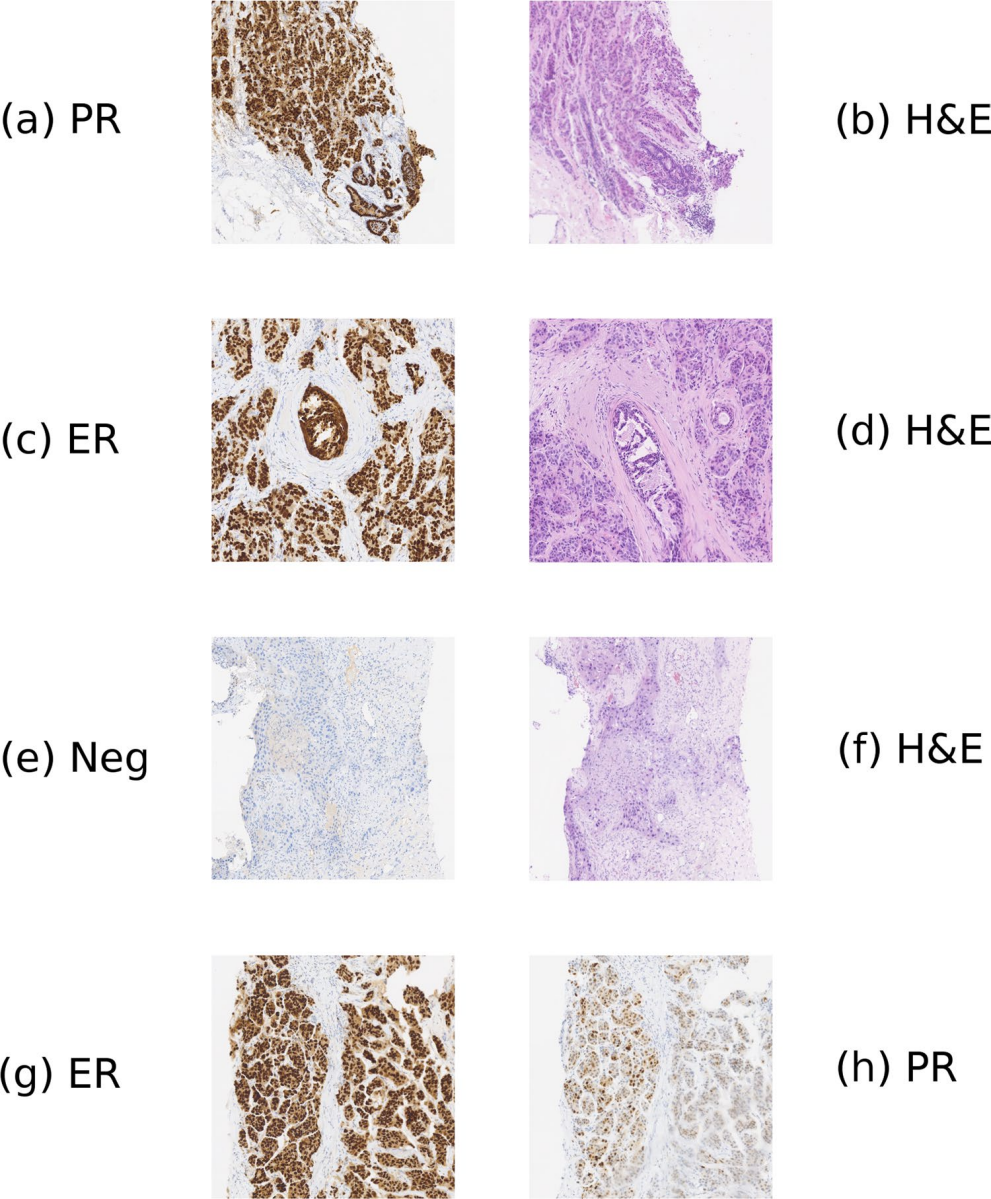
Examples of pairs of registered visual fields for a variety of stain types. Figure 2a,b shows the registration of an PR slide to an H&E slide. Figure 2c,d shows the registration of an ER slide to an H&E slide. Figure 2e,f shows the registration of an H&E slide to a negative control slide. Figure 2g,h shows the registration of an ER slide to a PR slide.

The results shown in Fig. 2 demonstrate that our registration algorithm is capable of good alignment of serial tissue sections, even in cases where there is not a perfect match of structures. In practice the thickness of serial sections in the tissue block can be relatively large, and thus some differences are expected between even adjacent sections. Consquently, in some cases partial matching of structures is needed and it is important that a registration algorithm is able to handle such situations.

**Figure 3:**
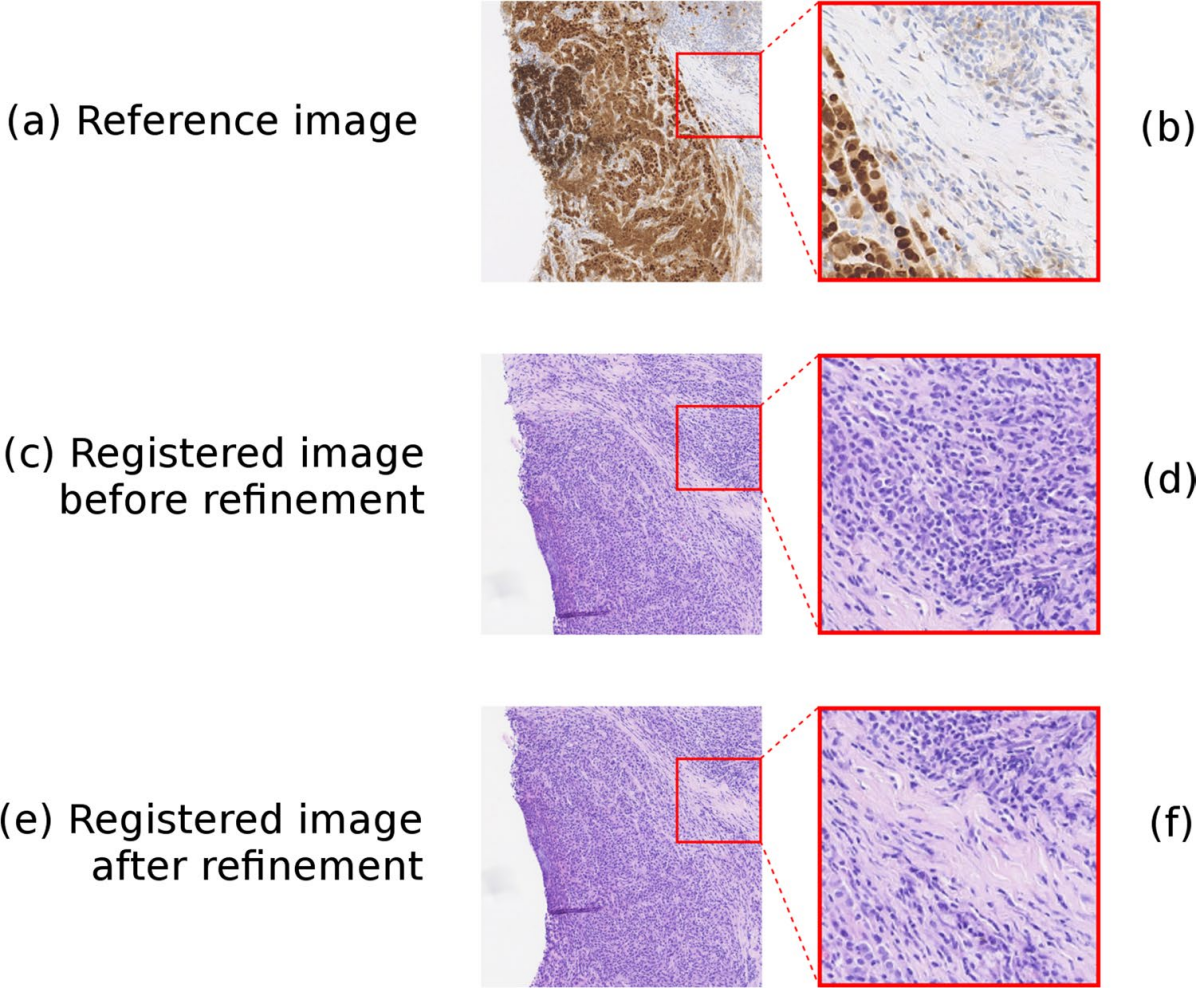
A demonstration of the registration algorithm’s refinement process. Figure 3a shows a visual field from the reference image, with a close-up of part of this region shown in Fig. 3b. Figure 3c shows the equivalent visual field from the registered image following approximate registration but before the registration refinement, and the equivalent close-up region is shown in Fig. 3d. Figure 3e shows the equivalent visual field from the registered image following registration refinement, and the equivalent close-up region is shown in Fig. 3f. It can be seen that the alignment of the two images is far better on a local level after refinement.

Tables 1 and 2 compare the results of registration with and without the refinement step on two different tissue types. The Chamfer distances given are the mean of the Chamfer distances, in pixels, between neighbouring sections following registration. It is clear that, while both provide good alignment of the tissue sections, the refinement step does bring the tissue into a better alignment for analysis at a higher resolution. A visual example of the registration before and after refinement can be seen in Fig. 3.

### Multi-IHC Scoring

In order to illustrate the benefits of simultaneous cross-slide analysis, we present a potential application of this work: automated scoring of sections stained with Estrogen Receptor and Progesterone Receptor (ER/PR), localised to an area of interest. In this case study, the slide images are segmented and registered, as detailed in the Methods section below, before continuing with the automated scoring algorithm. Regions of interest (ROIs) are chosen manually by an expert and it is only within these regions that scoring is performed. ROIs are annotated on an H&E slide, while scoring is performed on both ER and PR slides and therefore pre-registration is essential in order to find the equivalent ROI on the ER or PR section. Within the ROI a set of fields of view (FOVs) are chosen automatically as input for the scoring algorithm. By registering ER and PR slides it is possible to select a common set of FOVs as input to the scoring algorithm, which will guarantee that scoring is taking place on exactly the same regions of tissue for both stains.

**Figure 4:**
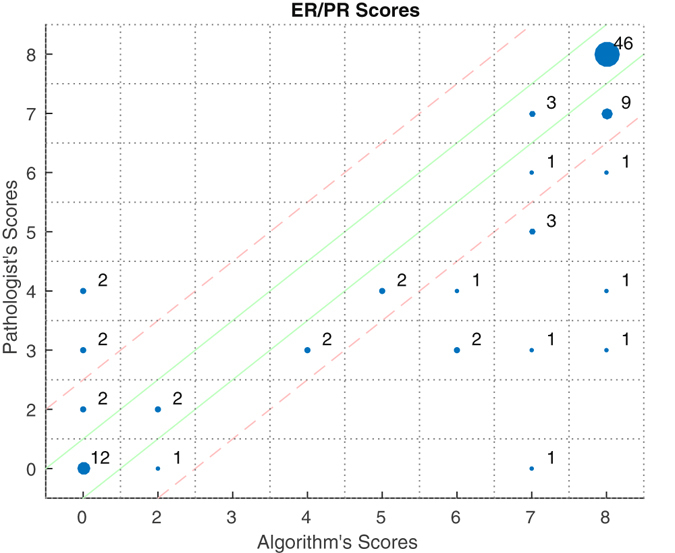
Scatter plot showing the results of the automatic ER/PR scoring, compared to the pathologist’s manual scores. Size of the dot is proportional to the number of cases with the given Pathologist-Algorithm score pair, the number of cases is also placed alongside the dot. All results between the solid green lines have the same score as the pathologist. All results between the dotted red lines are within one of the pathologist’s score.

For this case study, a set of 48 breast cancer cases, each with an H&E, ER and PR slide, were chosen. For each case, manual ER and PR scores were taken by the pathologist. Regions containing tumour were then digitally annotated on top of a single H&E slide image from each case. Following registration with the annotated H&E image, the system randomly selected 10 FOVs from the pathologist’s annotated area, each of size 1000 × 1000 pixels taken at the equivalent of a 20× magnification. The equivalent FOVs from the ER and PR sections are captured and scored. For a given stain, the mode of the 10 FOV scores is returned as its final score. In the event of a tie, five additional FOVs are selected and scored. Figure 4 shows a comparison between the computed scores and the pathologist’s scores on scatter plots for both ER and PR.

The mean absolute error of the automated scoring was found to be 0.42 for the ER sections and 0.89 for the PR sections, resulting in an overall mean error of 0.66. For the purpose of these calculations, scores of 0 were changed to 1 in order to ensure equal numerical spacing between the possible scores.

## Discussion

Registration of high resolution images, such as those in digital pathology, naturally lends itself to a multi-resolution strategy to image registration, owing to their large size and thus a large transformation space. A two-stage registration approach, such as that which has been employed in the proposed framework, is a practical implementation of this strategy. This is affirmed by the quantitative assessment of our registration approach, shown in Tables 1 and 2. The approximate registration method does require good tissue segmentation in order to produce boundaries that are able to be matched. This may be difficult to obtain in situations where tissue is very fragmented. It should be noted that this issue only relates to the approximate registration method and not the registration refinement. In such scenarios a different approximate registration could be used with the remainder of the existing framework unchanged. However this was not required for our data, as the segmentation algorithm was able to segment all breast and lymph node sections successfully.

The results of multi-IHC scoring, as shown in Fig. 4, demonstrate that the registration is suitable for use in cross-slide analysis, particularly for the area of IHC scoring. Furthermore, the high correlation between the scores of the pathologist and our algorithm is a clear indication that an automated system can achieve good agreement with an expert. This is true not just for the more common strongly positive and negative cases, which are arguably less difficult to score, but also for those that the pathologist scored in the middle of the Allred scale.

Automated digital pathology systems, such as the registration and multi-IHC analysis presented in this work, have the potential to complement, rather than replace, the pathologist’s existing practices. It has been suggested that providing the pathologist with additional objective information could lead to a number of potential long term benefits to their working practices, such as reducing the diagnostic time needed in certain cases^27^ and allowing for better supported decision making^28^. This second point is particularly important when one considers the training of future pathologists, for whom the additional information may serve as a key learning aid. Additionally, this may help balance against external factors that result in both inter- and intra-observer variability in pathologists’ judgement^27, 29, 30^.

It should also be recognised that registration has value outside of pre-processing for downstream analysis tasks, such as scoring. The ability to view and manipulate multiple whole-slide images at the same time, through the use of registration, could also be a vital component of a standalone slide viewing tool. While its primary function is multi-IHC stain analysis, the Hyper-Stain Inspector software also contains such functionality. Simultaneous slide manipulation could play an important role in the training of future pathologists, as it can allow a trainee to directly compare tissue morphology and different stain expressions across entire slides interactively, rather than having to rely on textbook examples.

To conclude, cross-slide analysis plays an important role in traditional pathology and is likely to continue to do so in the realm of digital pathology. A significant advantage of digital pathology lies in the potential for automation of tasks which are currently completely manual, but this cannot be achieved for cross-slide analysis without first realigning the set of serial slide images. Systems that combine automated registration with downstream algorithmic analysis, as demonstrated with the Hyper-Stain Inspector, are therefore likely to play a key role in areas such as IHC stain co-expression and scoring.

## Methods

### Tissue Boundary Registration

#### Segmentation

The proposed algorithm determines the initial registration using the external boundaries of the two tissue sections. The sections must therefore be segmented from the background before the registration can take place. We employ a tissue segmentation method based upon the entropy filter response^31^. Areas of the glass slide without tissue are mostly a single colour, with very little variation, and as such regions with no tissue are likely to have a very low filter response. We are therefore able to generate an initial set of tissue boundaries by applying Otsu thresholding^32^ to the filter response.

The resultant set of detected tissue regions may include many erroneous detections, such as slide labels, smudges, and other physical slide artefacts. When these artefacts overlap a tissue section it may also interfere with the quality of the segmentation. We use a supervised SVM classifier, trained with a Local Binary Pattern (LBP) texture feature^33^, to eliminate many of these falsely segmented regions. Classification is performed on 64 × 64 pixel windows within the original segmented region, with fields of view (FOVs) extracted at twice the resolution of the initial segmentation. Windows that the classifier rejects are removed from the silhouette of the detected section. If a majority of a section’s windows are rejected by the classifier, then it will also be removed from the output set of segmentations.

**Figure 5:**
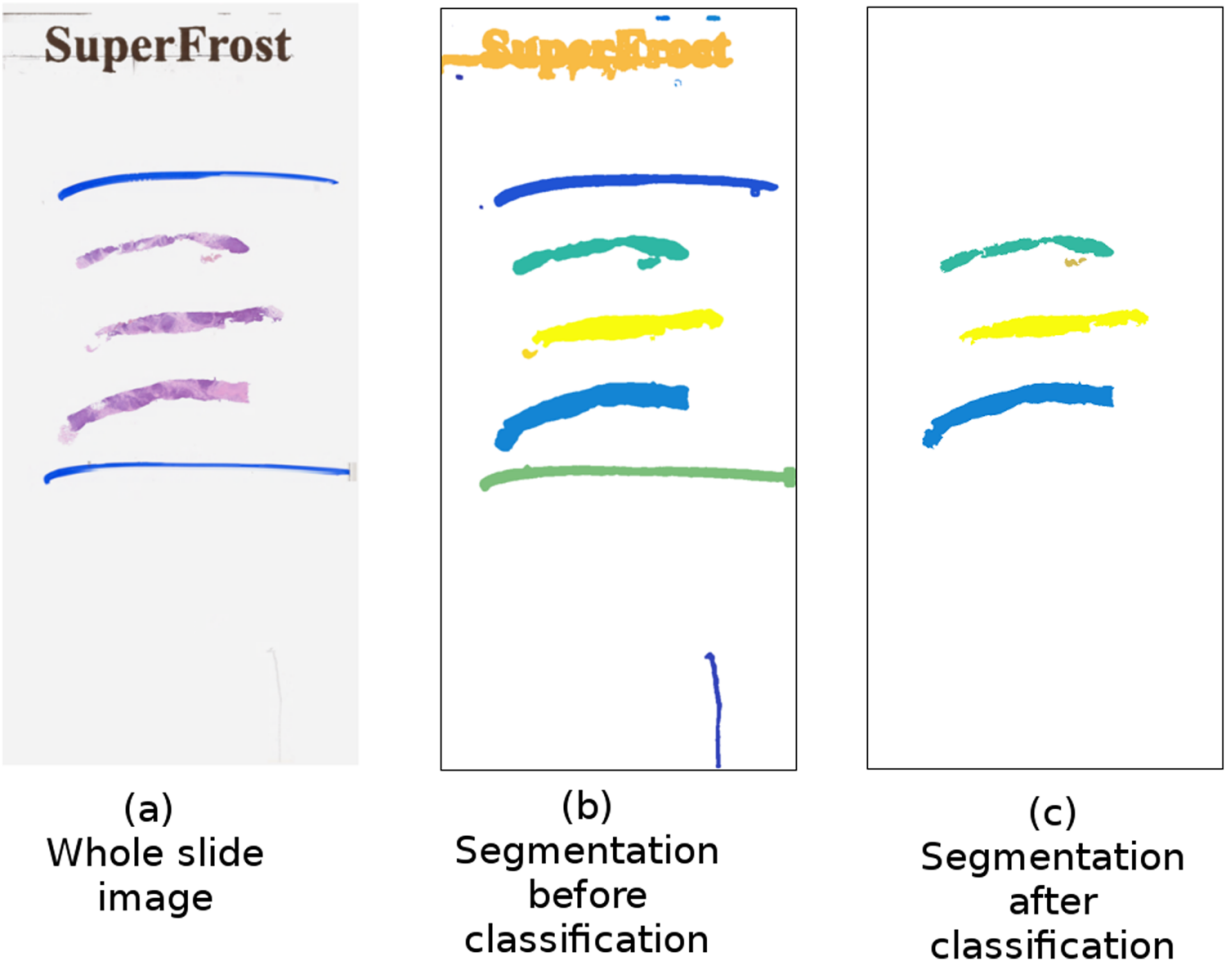
An example of the results of tissue segmentation. Figure 5a shows the original Whole Slide Image, Fig. 5b shows the initial results of segmentation, Fig. 5c shows the final tissue segmentation after tissue classification has been performed.

**Figure 6:**
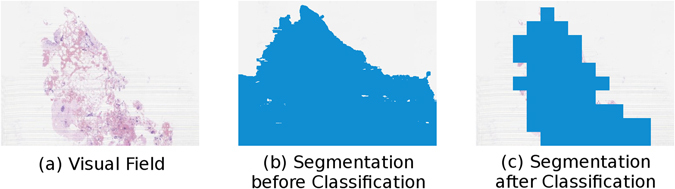
An example of the results of tissue segmentation on a region containing artefacts. Figure 6a shows the visual field, with visible artefacts on the left and right sides of the tissue. Figure 6b shows the initial results of segmentation, which has false captured the artefacts as tissue. Figure 6c shows the final tissue segmentation after tissue classification, in which the artefactual regions have been removed.

An example of the result of segmentation is shown in Fig. 5. Note that while the sections have been successfully segmented, segmentations for the slide’s label and the pen marks above and below the sections have been identified and removed by the tissue classifier. Figure 6 shows an example of the result of segmentation on a region of a slide containing artefacts. In this example it can be seen that, although the region containing the artefacts is initially segmented as tissue, the tissue classifier is able to identify these regions and remove them from the final segmentation.

Each remaining detected region is converted into a set of points, defining the external boundary of the region in question. The boundary is then parametrised into *N* equally spaced points, this step is important as the registration method requires the boundaries to have an equal number of points. For this work we use *N* = 2000 points, which is sufficent to describe the boundary in detail. The set of points is then used for approximate registration. Prior to registration, one of the detected tissue sections must be chosen as the reference, to which the remainder of the slides in the case will be registered. Currently the reference is selected by the user within the Hyper-Stain Inspector software. For a set of sections taken from a single block, it is normally the case that each slide will have a similar arrangement of tissue sections. In spite of this, is still important to select a specific reference section for registration, because the required transformation for alignment may vary slightly for each section.

#### Approximate Registration

We model the initial approximation as a globally rigid transformation, specifically a transformation composed of only translations and rotations. Since curvature is invariant to both translation and rotation, we align the boundaries of tissue sections by matching their points of locally maximal curvature^18^. Curvature is a feature that can vary depending on the resolution, or the level of detail, at which the curve is observed. Therefore, in order to make a thorough comparison of curvature, a multi-resolution representation of curvature is needed.

Curvature Scale Space (CSS)^19^ is a representation of a polygon’s curvature across a set of decreasing resolutions, simulated by smoothing the polygon with Gaussian filters of increasing bandwidth. Specifically, the CSS representation of a polygon is an *n* × *m* image, where each row represents the *n* points of the polygon at a particular resolution, and each column represents a point of the polygon across the *m* resolutions. For a boundary *P* = *p*_1_, …, *p*_*n*_, where *p*_*i*_ = (*x*_*i*_, *y*_*i*_) is a point on the boundary, and a set of *m* resolutions *σ* = *σ*_1_, …, *σ*_*m*_, we define the CSS image as follows:

2 $${P}_{j}=G(P,{\sigma }_{j})\quad j\in \{1,2,\ldots ,m\}$$

3 $${\kappa }_{i,j}=\frac{{x}_{i,j}^{^{\prime} }{y}_{i,j}^{^{\prime\prime} }+{y}_{i,j}^{^{\prime} }{x}_{i,j}^{^{\prime\prime} }}{{({x}_{i,j}^{^{\prime} 2}+{y}_{i,j}^{^{\prime} 2})}^{\frac{3}{2}}}$$

4 $$CSS(i,j)=(\begin{array}{ll}1 & {\rm{if}}\,{\kappa }_{i,j}\,{\rm{is}}\,{\rm{a}}\,{\rm{local}}\,{\rm{maxima}}{\rm{.}}\\ 0 & {\rm{otherwise}}{\rm{.}}\end{array}$$

where *G*(*P*, *σ*_*j*_) is a Gaussian filter with standard deviation *σ*_*j*_ applied to boundary *P*, *κ*_*i*,*j*_ is the curvature at *P*_*i*,*j*_ = (*x*_*i*,*j*_, *y*_*i*,*j*_), the *i*th point of boundary *P*_*j*_. *x*′ and *x*″ denote the first and second derivatives of *x*.

Two changes are made to the original CSS formulation^19^. First, the number of resolutions in the CSS representation is sampled exponentially, rather than at constant intervals. The reason is that the Gaussian smoothing is a computationally costly operation, and thus it is beneficial to reduce the number of low resolution boundaries we need to compute. The difference between boundary produced by adjacent *σ*s is likely to be very small, and thus the curvature values will be almost the same. By using a smaller set of *σ*s spaced exponentially, we still capture the variation of the maxima at different resolutions but with far fewer samples.

The second change is in the CSS formulation itself. In the original definition, the CSS image has a value of 1 at zero-crossings of curvature, or rather inflection points of the boundary. In our images, these points were found to be unstable and not consistent across serial sections. This is because the most common inflection points on a tissue boundary occur where there is a series of points in an approximately straight line. In such a situation the curvature will gently fluctuate between positive and negative many times, but these are relatively minor features of the tissue and are unlikely to be consistent across all serial sections. Curvature maxima, however, are major features of the boundary and, therefore, are unlikely to change drastically across serial sections, making them ideal for registration.

The points of the curvature maxima will be at similar locations on each boundary for all but the highest resolutions. Thus, it should be possible to find a close matching between the sets of curvature maxima from the two boundaries, allowing us to generate the best-fit rigid transformation for registration. Matching between maxima can be thought of as an assignment problem and is solved using the Hungarian algorithm^34^.

### Local Refinement

The refinement step can be coarsely divided into two main sub-components, tissue structure detection and tissue structure registration. The detection phase is designed to highlight parts of the tissue that are distinguishing features of the FOV. Such tissue features are likely to persist across many sections, and consequently be good features for aligning serial sections. Two types of tissue features chosen for this work are nuclei clusters and fat. Other tissue structures, such as glands, were also considered, but were ultimately not included due to their low density in many tissue types. While additional structures may improve registration in certain scenarios and for certain types of tissue, it was found that for our cases the two chosen features were sufficient for high quality registration.

#### Tissue Feature Detection

Pockets of fat in the tissue do not stain and have no visible texture on the slide. As a consequence, they appear almost identical to empty glass. Fatty regions can, however, be distinguished from empty slide by the presence of thin pieces of connective tissue on the edge between fatty regions. Therefore, we first identify potential fatty regions by thresholding on the luminance of the image, the threshold value is estimated from pixels sampled from a region of the slide that we know to be empty. Pockets of fat are selected as the connected regions above the threshold which are surrounded by a connected set of pixels that are below our chosen threshold, which are likely to correspond to the connective tissue around the pocket of fat.

By contrast, nuclei clusters are detected within the Haematoxylin stain channel, and thus we first apply a method of automatic stain separation^35^ to extract the Haematoxylin channel from the RGB image. Thresholding is applied to the separated stain channel, producing a set of connected components, coarsely corresponding to the individual nuclei. Rather than try to match on the level of individual nuclei which, for most part, are unlikely to be present in both tissue sections, we instead group them into clusters. The general arrangement of nuclei is likely to persist across the tissue for some time, and therefore alignment of collections of nuclei does not have the same problems associated with nucleus-to-nucleus matching. Density based clustering is applied to the detected nuclei using the DBScan algorithm^36^.

**Figure 7:**
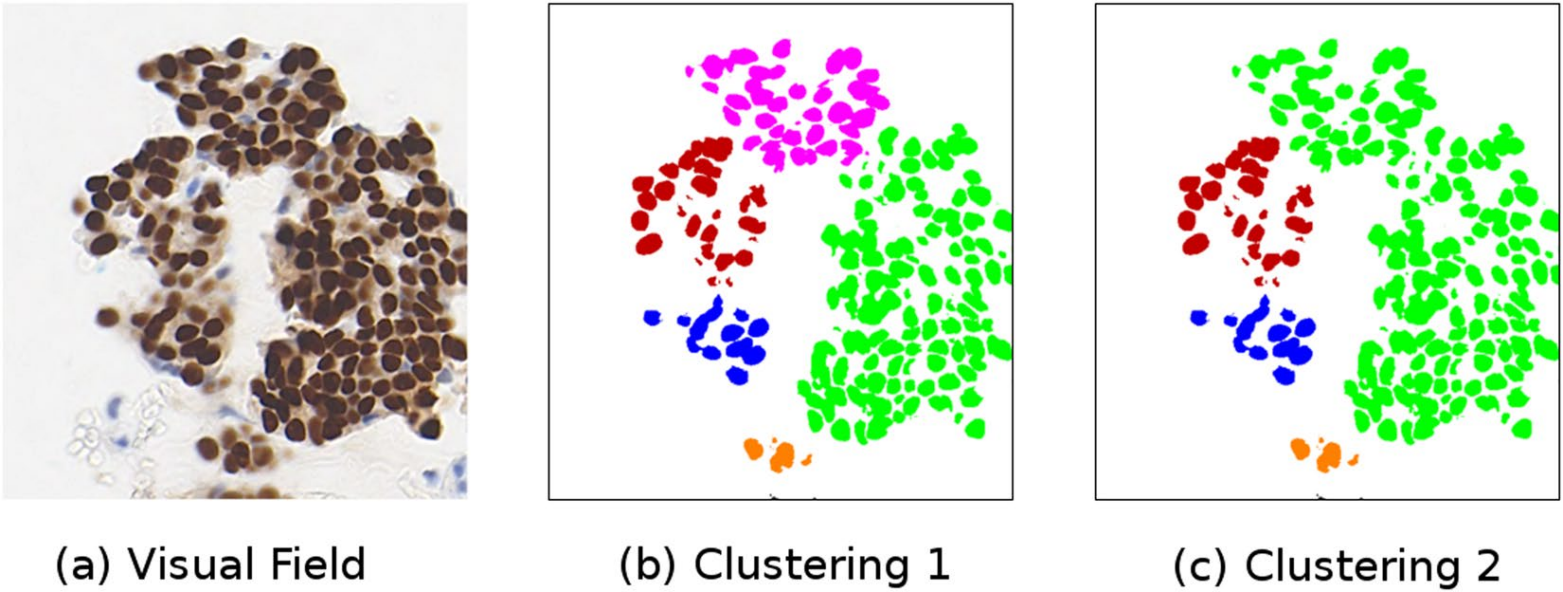
An example of two potential nuclei clusterings. Figure 7a shows the original image, Fig. 7b shows one potential clustering of the detected nuclei, Fig. 7c shows a different potential clustering of the detected nuclei.

In tissue with densely packed nuclei, the boundaries between each cluster may be difficult to identify with any precision, and thus there are several possible clusterings that could be generated. Consider the case shown in Fig. 7, where the collection of nuclei could be clustered into one or two groups, depending on the parameters used. This problem is confounded in the case of serial sections, where the slight differences in the arrangement of nuclei may also produce slightly difference clusterings.

**Figure 8:**
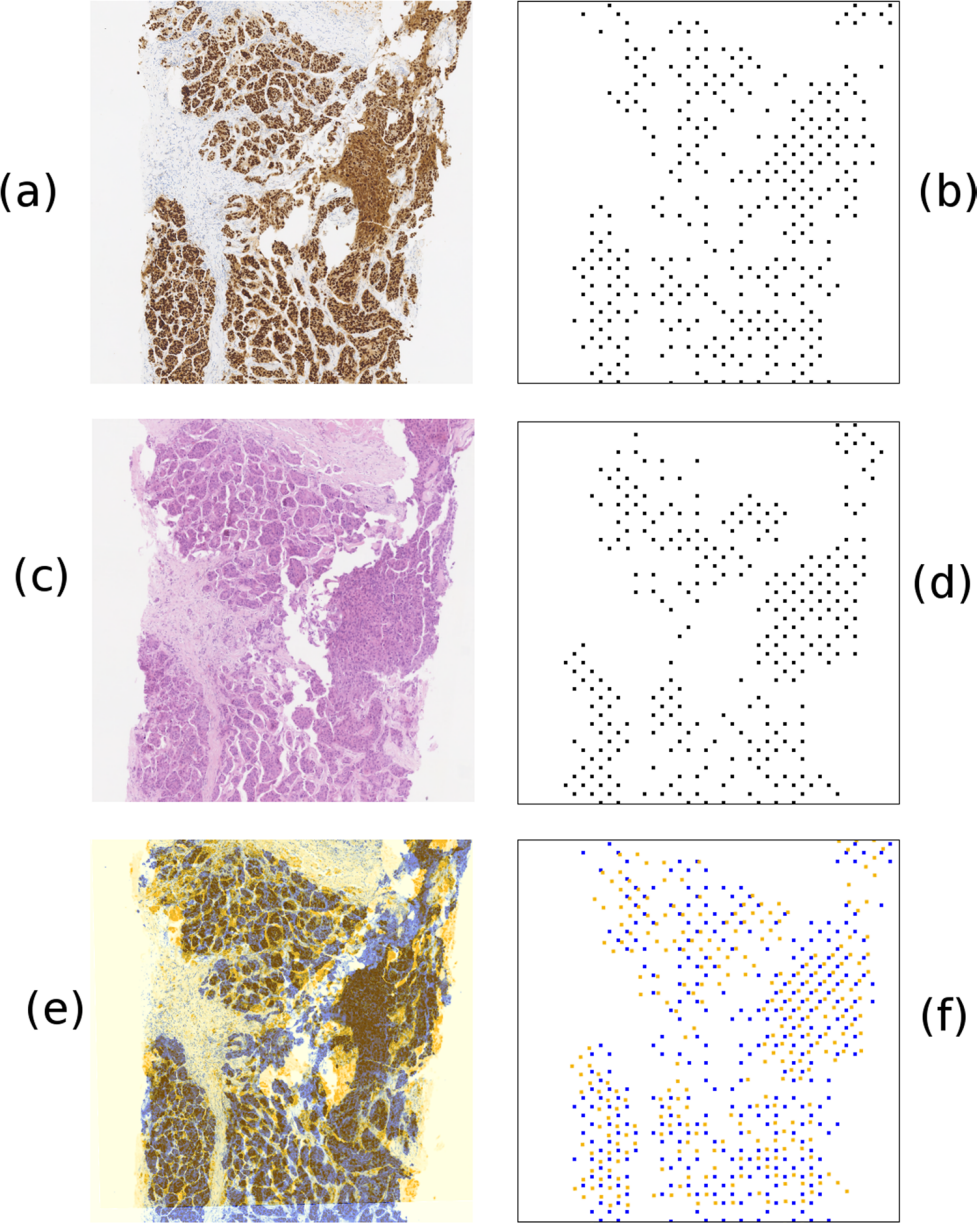
A demonstration of the nuclei cluster detection on two breast tissue slides. Despite many differences in the images, the extracted features are arranged very similarly and register the image well. A visual field from an ER stained slide is shown in Fig. 8a, with its nuclei cluster detection results shown in Fig. 8b. A visual field from an H&E stained slide is shown in Fig. 8c, with its nuclei cluster detection results shown in Fig. 8d. The registered visual fields are shown as a false colour image in Fig. 8e, with the ER image in blue and the H&E image in orange. The positions of the detected nuclei clusters following registration are shown in Fig. 8f, ER detections are shown in blue and H&E detections are shown in orange.

This problem can be addressed by allowing for partial matches between clusters, which is achieved by converting the cluster to a set of equally spaced points occupying its entire area with a specified point density. Our chosen point density of 0.004 points/*μm* ^2^, equivalent to 0.01 points/pixel at our chosen resolution, was found to be sufficient to describe the nuclei arrangement of the nuclei cluster. An illustration of the clustering approach is shown in Fig. 8. This figure also demonstrates how matching can be achieved when clustering differs between sections.

#### Registration of Salient Histological Features

Registration of features is performed in a partial matching framework. As described above, the term feature refers to either a single detected pocket of fat or a point from a nuclei cluster. Each feature has an associated (*x*, *y*) coordinate pair: the position of the feature’s centroid. If the tissue structures of the serial sections are sufficiently similar then the optimal registration will produce a close alignment of the features, and consequently a close alignment of their centroids. We can therefore consider this step as a point set registration task. We apply a Coherent Point Drift (CPD)^13^ approach to find the optimal point assignment, restricting the search to rigid alignments. An approach using an Iterative Closest Point (ICP)^11^ algorithm was also considered. However, ICP was found to produce sub-optimal local minima on some cases where the initial registration was poor.

CPD models the points to be registered as the centroids of a Gaussian Mixture Model (GMM), while the reference set of points are treated as points within the GMM. The intention is to find the transformation parameters that coherently transform the points such that the GMM’s posterior probability is maximised, which should be achieved when the two point sets are well registered. Expectation Maximisation is used to find the best alignment parameters. The GMM probability density function for a point *z* is traditionally defined as

5 $$p(z)=\sum _{m=1}^{M+1}P(m)p(z|m)$$

where *M* is the number of points in the registered point set, $P(m)=\frac{1}{M}$ are the membership probabilities, and *p*(*z*|*m*) is a Gaussian distribution with centroid at point m.

Modifications were made to the original formulation of CPD to address issues that may arise if an accurate match of the features is not possible. In cases where the distance between sections within the block is relatively large, there may be very little similarity between the two sections’ internal tissue structures. This means that, while the shape of the sections may be similar, their detected features are likely to be very different, and therefore registration in this way will not be possible. In such a scenario the results of approximate registration would be preferable to the alignment of dissimilar tissue features. This situation may also arise if the sets of tissue features are very small, which may occur when registering small regions of tissue at high resolution.

It is assumed that corresponding features are within a certain distance of each other following approximate registration. In view of this, we define a replacement for CPD’s *p*(*z*|*m*) term as follows:

6 $$q(z|m)=p(z|m)d{(z,\tilde{z})}^{\beta }$$

where *z* is the current position of the given point and $\tilde{z}$ is its position following approximate registration. *β* is a weighting parameter on the function d, which can be altered to adjust the balance between the approximation and the refinement; in our experiments a value of *β* = 1 was chosen.

7 $$d(z,\tilde{z})=(\begin{array}{ll}\frac{\sqrt{{\rm{\max }}({\delta }^{2}-{\Vert z-\tilde{z}\Vert }^{2},\mathrm{0)}}}{\delta } & {\rm{if}}\,{\rm{the}}\,{\rm{features}}\,{\rm{are}}\,{\rm{of}}\,{\rm{the}}\,{\rm{same}}\,{\rm{type}}{\rm{.}}\\ 0 & {\rm{otherwise}}\mathrm{.}\end{array}$$

is a term designed to restrict matching of features to only those features that are within *δ* of each other, following approximate registration. It was found that *δ* = 33 *μm*, equivalent to 15 pixels at our chosen resolution, is sufficient to produce an accurate registration. The condition that features must be of the same type is included to avoid the matching of unrelated tissue features.

Finally, phase correlation is performed on the aligned images. It was found that the alignment produced at this stage is sometimes a small translation away from the actual alignment. This is likely to exist because the points chosen for the localised registration are an estimated centre of the associated structure, which may be offset from the true location slightly. If the phase image’s peak is inside the central 20%, or rather if the estimated translation offset is less than 10% of the image size in any direction, then the image is shifted accordingly. Phase correlation was not used wthin the earlier stages of the framework due to concerns of robustness. It has been shown that bright images often do not register well with darker images^37^, a scenario that is comparable to the registration of a WSI with weak staining to a WSI with strong staining.

Examples of registration before and after the refinement step are shown in Fig. 3. Prior to the correction step it can be seen that, while the two sections have been aligned with some success by the approximate method, the co-registered sub-regions outlined in red do not correspond to the same area of tissue. Following the correction step, it can be seen that the sections have been aligned more precisely and the two sub-regions now correspond to the same part of the tissue. It should be noted that this adjustment is equalent to approximately 1 mm of translation, a fairly minor adjustment in a global sense but one which is key for closely registering the finer details of each section.

### Ethical Approval Statement

Ethical approval for this study was granted by the National Research Ethics Service - Dulwich Committee 12/LO/0993.

### Data Availability Statement

The datasets analysed during the current study are available from the corresponding author on reasonable request.
